# Refractory celiac disease and its mimickers: a review on pathogenesis, clinical-pathological features and therapeutic challenges

**DOI:** 10.3389/fonc.2023.1273305

**Published:** 2023-11-07

**Authors:** Federico Scarmozzino, Marco Pizzi, Filippo Pelizzaro, Valentina Angerilli, Angelo Paolo Dei Tos, Francesco Piazza, Edoardo Vincenzo Savarino, Fabiana Zingone, Matteo Fassan

**Affiliations:** ^1^ Surgical Pathology and Cytopathology Unit, Department of Medicine-DIMED, University of Padua School of Medicine, Padua, Italy; ^2^ Gastroenterology Unit, Department of Surgical, Gastroenterological and Oncological Sciences -DISCOG, University of Padua School of Medicine, Padua, Italy; ^3^ Hematology & Clinical Immunology Unit, Department of Medicine-DIMED, University of Padua School of Medicine, Padua, Italy; ^4^ Veneto Institute of Oncology, IOV-IRCCS, Padua, Italy

**Keywords:** coeliac disease, refractory coeliac disease, enteropathy-associated T-cell lymphoma, gastrointestinal lymphomas, differential diagnosis

## Abstract

Refractory celiac disease (RCD) and enteropathy-associated T-cell lymphoma (EATL) are rare, yet severe complications of celiac disease (CD). Over the last decades, several studies have addressed the biology and clinical-pathological features of such conditions, highlighting unique disease patterns and recurrent genetic events. Current classification proposals identify two forms of RCD, namely: (i) type 1 RCD (RCD-I), characterized by phenotypically normal intra-epithelial lymphocytes (IELs); and (ii) type 2 RCD (RCD-II), featuring phenotypically aberrant IELs. While RCD-I likely represents a gluten-independent dysimmune reaction against small bowel epithelial cells, RCD-II is better considered an *in situ* aggressive T-cell lymphoma, with high rates of progression to overt EATL. The diagnosis of RCD and EATL is often challenging, due to misleading clinical-pathological features and to significant overlap with several CD-unrelated gastro-intestinal disorders. Similarly, the treatment of RCD and EATL is an unmet clinical need for both gastroenterologists and hematologists. Moving from such premises, this review aims to provide a comprehensive view of RCD and EATL, specifically considering their pathogenesis and the many still open issues concerning their diagnosis and clinical management.

## 1 Introduction

Celiac disease (CD) is a T-cell mediated small intestinal autoimmune-like disease triggered by ingestion of gluten proteins in genetically susceptible individuals. CD is one of the most common autoimmune diseases, affecting approximately 1% of the Western population. Although CD can occur at virtually any age, most cases are diagnosed in children and young adults (1). Almost all CD patients carry one or both of the human leukocyte antigens (HLA) DQ2 and DQ8. Rare HLA-DQ2/DQ8-negative cases are positive for HLA-DQ7.5 (<1% of patients) (2, 3).

The clinical presentation of CD is broad, ranging from fully asymptomatic cases to very morbid conditions. Most symptoms are related to malabsorption, micronutrient deficiency and failure to thrive, as a result of intestinal mucosa damage by gluten-induced dysimmunity. Non-classical presentations can involve extra-gastro-intestinal (GI) sites and include neurological symptoms, endocrinopathies, cutaneous lesions, osteopenia and changes in reproductive function (4, 5). Finally, long-lasting and/or untreated CD can undergo severe complications including small bowel adenocarcinoma and an aggressive form of peripheral T-cell lymphoma, referred to as enteropathy-associated T-cell lymphoma (EATL) (6).

The diagnosis of CD rests on a combination of serologic testing and histological findings (1). Serological diagnosis requires the documentation of CD-specific auto-antibodies (auto-Ab) of either IgA or IgG class (*i.e.* anti-deamidated gliadin peptide [anti-DGP] auto-Ab; anti-tissue transglutaminase [anti-tTG] auto-Ab; anti-endomysial Ab [EMA]). Testing for IgA auto-Ab is routinely performed in the diagnostic workup of all suspected CD patients, while IgG auto-Ab are mainly tested in cases with selective IgA deficiency (5).

Duodenal biopsy should be performed in all adult patients with suspected CD and positive CD-specific auto-Ab. In cases with negative serology, histological examination is recommended only if clinical data are highly suspicious for CD. In children, duodenal biopsy can be avoided if high titers of IgA anti-tTG auto-Ab and EMA are detected (7). A minimum of 4 biopsy samples are required for histological evaluation (2 biopsies from the duodenal bulb and 2 from the second duodenal portion) (1). Biopsy samples should be correctly orientated (possibly on filter paper) and should contain ≥3–4 consecutive villi-crypt units (7). Histologically, the diagnosis of CD requires the documentation of increased intraepithelial lymphocytes (IELs) at duodenal biopsy (≥25 lymphocytes/100 epithelial cells) with variable degrees of villous atrophy and/or crypt hyperplasia. IELs typically disclose a ‘crescendo pattern’, whereby lymphocytes mostly locate in the upper two thirds of villous epithelium. Based on the severity of mucosal changes, CD is histologically graded according to Corazza-Villanacci and Marsh-Oberhuber schemes (8–11).

Once the diagnosis of CD is established, the only proven treatment consists in strict adhesion to life-long gluten-free diet (GFD) (7). In most cases, GFD leads to complete remission of clinical, serological and histological alterations, although this may take months to years to occur (12). Poor response to GFD is mainly due to poor patient compliance and/or inadvertent food contamination with gluten (13–16). In rare instances, however, GFD failure depends on CD-intrinsic factors, which are responsible of so-called refractory celiac disease (RCD).

According to international consensus reports, RCD is defined as any CD with clinical and histological unresponsiveness to ≥12 months of strict GFD (17, 18). This broad definition encompasses different types of RCD, with variable biological, clinical and prognostic features. As such, the diagnosis and sub-categorization of RCD is often challenging, and its management is still an unmet clinical need. Moreover, the boundaries between RCD and EATL are often blurred, likely as a result of the biological continuum between these entities.

Moving from such premises, this review aims at providing a comprehensive view of the pathogenesis and clinical-pathological features of RCD and EATL, specifically focusing on the most recent biological achievements and on their clinical implications.

## 2 The spectrum of RCD: classification and clinical features

### 2.1 Classification and epidemiology of RCD

According to a systematic review published in 2016, RCD has a prevalence of 0.3-0.4% and a cumulative incidence of 1-4% among CD patients (19). RCD usually affects adult to elderly patients, with most cases being diagnosed between 40 and 60 years of age (20, 21). Compared to GFD-responsive cases, RCD has a longer interval between onset of enteropathy-related symptoms and CD diagnosis, suggesting a direct role for protracted gluten exposure in the pathogenesis of RCD (22).

In the last decades, the incidence of RCD has progressively decreased, possibly as a result of timelier diagnoses of CD and of wider availability of gluten free products (22). Besides gluten exposure, the main risk factors for RCD include male gender and old age at diagnosis, classical symptomatic CD at presentation, negativity for CD-related auto-Ab at the time of diagnosis (23, 24).

RCD can be classified into two subtypes, depending on the immunophenotype of intraepithelial lymphocytes (IELs): (i) RCD type I (RCD-I), characterized by normal (surface CD3 [sCD3]+/CD8+) IELs; and (ii) RCD type II (RCD-II), characterized by phenotypically aberrant (sCD3-/cytoplasmic CD3 [cCD3]+/CD8-) IELs. In most studies, RCD-I occurs one decade earlier than RCD-II (mean age at diagnosis: 40-50 *versus* 50-60 years) (20–22, 25). . The proportion of RCD subtypes is inconsistent across series and remains largely undefined (26). The biological differences between RCD-I and RCD-II subtend relevant differences in terms of prognosis and treatment options.

### 2.2 Clinical and laboratory features of RCD

Clinically, RCD-I and RCD-II present with symptoms of untreated CD, including long-lasting diarrhea, abdominal pain, weight loss, fatigue and malaise (21, 27). Symptom burden is usually worse in RCD-II, due to extensive bowel involvement (28) and mucosal ulcerations (20). Concurrent autoimmune/dysimmune diseases are frequently reported (*e.g.* Hashimoto’s thyroiditis; microscopic colitis; autoimmune hepatopathies), being slightly more common in RCD-II than RCD-I (20). Systemic symptoms (*i.e.* drenching night sweats, fever, and weight loss), small bowel strictures and occlusions are hallmark of EATL progression (20).

Laboratory tests typically disclose anemia, multiple vitamin deficiencies, chronic hyper-transaminasemia (21). The latter correlates with intestinal mucosal damage (20) more frequently reported in RCD-II than RCD-I (70% versus 21% of cases) (29). Although most patients have negative CD-specific antibodies at the time of RCD, positive auto-Ab does not necessarily exclude the diagnosis (20, 27). Compared to uncomplicated CD, RCD-I/RCD-II usually disclose higher Chromogranin A (CgA), β2-microglobulin (B2M) and lactate dehydrogenase (LDH) serum levels (30). B2M and LDH likely parallel lymphoid cell expansion, while CgA correlates with neuroendocrine cell hyperplasia (CgA) (31). As such, serum CgA, B2M and LDH testing may serve as cost-effective strategies for an early diagnosis of RCD.

### 2.3 Diagnostic workup of RCD

**Figure 1:**
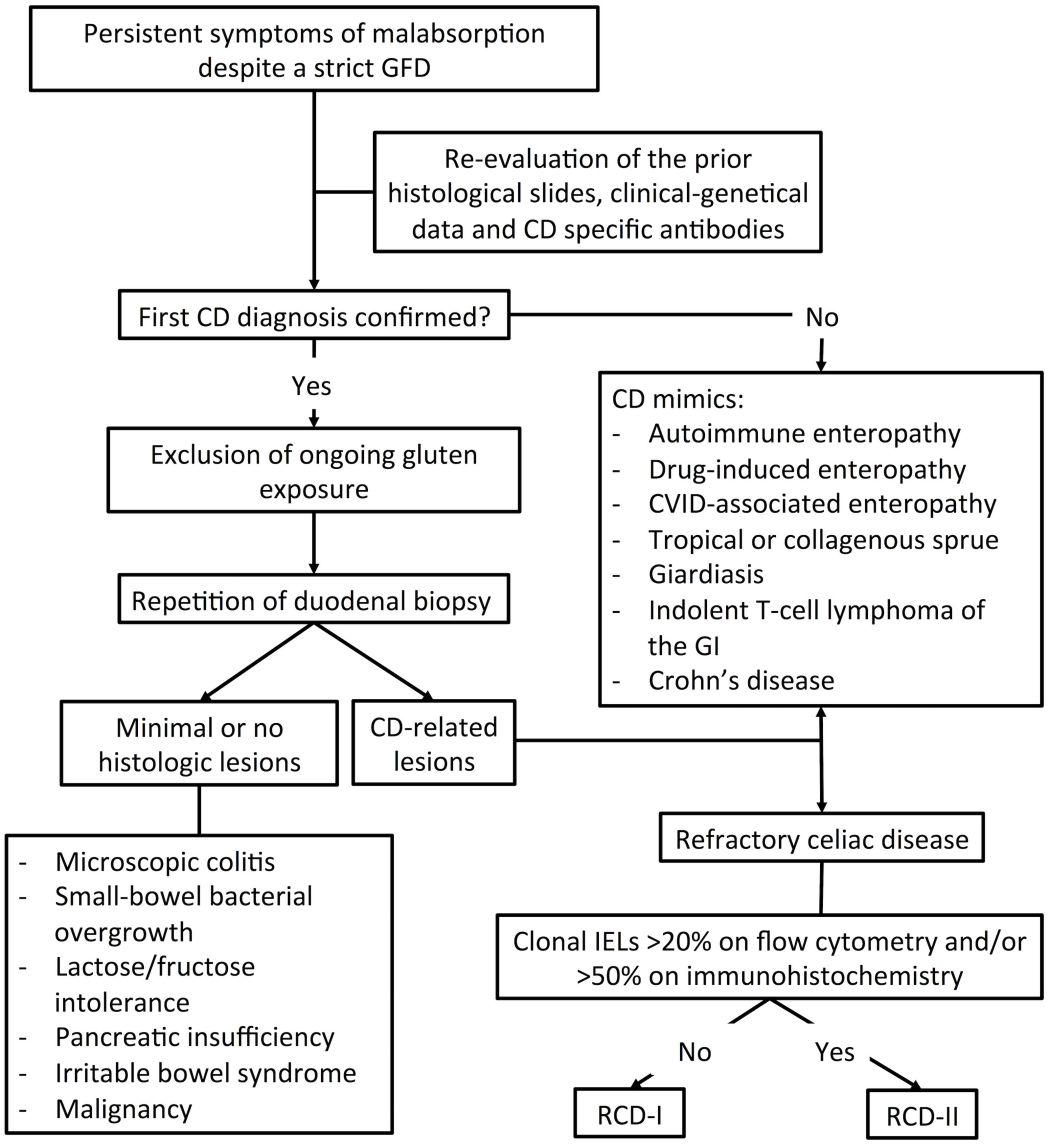
Diagnostic workup for Refracrory Celiac Disease (RCD).

The diagnosis of RCD is often challenging and, in most cases, one of exclusion (**Figure 1**). The first step in the diagnostic workup is confirming the original diagnosis of CD. This is usually achieved by re-evaluation of clinical, genetic and histological data, as well as by confirmation of CD-specific auto-Ab (17, 32).

Once the diagnosis of CD is confirmed, adherence to GFD should be carefully assessed. By far, the most common cause of symptom persistence in CD is ongoing gluten exposure with diet. This is documented in roughly 50% of patients with putative RCD (13–15) and should be investigated by dietary interview, testing for CD-specific auto-Ab and/or for gluten peptides in urine/stool samples (32–34). Persistence of anti-tTG auto-Ab and/or EMA should specifically raise concern of ongoing gluten exposure (20, 21).

If adherence to GFD is proven, endoscopic exams and biopsies should be repeated (2). The documentation of CD-related lesions suggests ongoing gluten exposure, RCD or any of its mimickers associated to villous atrophy (see paragraph 4.1) (2). However, if minimal or no microscopic changes are observed, other causes of abdominal discomfort should be considered (30), such as microscopic colitis, small-bowel bacterial overgrowth (SIBO), lactose intolerance, pancreatic insufficiency, or irritable bowel syndrome (32, 35, 36) (**Figure 1**). According to the latest ESGE guidelines, both a standard esophagogastroduodenoscopy (EGDS) and capsule endoscopy (CE) should be performed in non-responsive CD-patients after excluding gluten ingestion (37). CE allows exploring the small bowel beyond the Treitz ligament, where lesions suggestive of RCD-II and EATL often locate. In particular, the finding of ulcerative jejunitis and/or of large (≥1 cm) ulcerations should specifically raise concern for RCD-II or EATL (20). Further device-assisted enteroscopy (DAE) allows to obtain tissue samples for accurate diagnosis and subsequent treatment (37). Therefore, both standard EGDS and CE/DAE are crucial for patients suspected of having RCD. In all suitable cases, CE should be preceded by small bowel-directed radiological studies (*e.g.* CT or MR enterography) to detect intestinal strictures or wall thickening that may hamper endoscopic evaluation. Imaging studies may also aid disclosing abdominal masses and mesenteric lymphadenopathies (27, 38), heralding RCD-II and EATL-related complications (**Table 1**). Splenic atrophy is a further finding, especially in cases of RCD-II and EATL (39).

**Table 1:** Distinguishing features of CD, RCD-I, RCD-II, and EATL.

	CD	RCD-I*	RCD-II	EATL
**B symptoms**	–	–	-/+	+
**Small bowel occlusions**	–	–	–	+
**Abdominal masses**	–	–	–	+
**Mesenteric lymphadenopathies**	–	–	–	+
**Aberrant IEL morphology**	–	–	-/+	+
**Aberrant IEL phenotype**	–	–	+	+
**Proliferation index**	Low	Low	Low	High
**CD30 expression**	–	–	–	+
**Infiltration of the LP**	–	–	+ (minimal)	+ (massive)
**Treatment**	Gluten-free diet	Corticosteroids +/- Immunosuppression	Corticosteroids +/- Chemotherapy +/- ASCT	Chemotherapy + ASCT
**5-year overall survival**	≈100%	80-95%	44-58%	11-20%

*RCD-I is distinguished from CD on clinical grounds only (i.e. persistence of malabsorption and villous atrophy after ≥12 months of strict gluten-free diet)

CD, Celiac disease; RCD-I, Refractory celiac disease type I; RCD-II, Refractory celiac disease type II; EATL, Enteropathy-associated T-cell lymphoma; IELs, Intraepithelial lymphocytes; LP, Lamina Propria; ASCT, Autologous stem cell transplantation.

Endoscopic studies should be integrated with biopsy sampling and histological re-evaluation. A definite diagnosis of RCD can be made only when CD-related lesions are documented and all CD-mimickers are confidently excluded.

### 2.4 Treatment and prognosis of RCD

The treatment of RCD is challenging and varies depending on disease subtype. Nutritional support and corticosteroids (*i.e.* open capsule budesonide or prednisone) are the first line therapies for RCD-I, being associated with clinical improvement in most cases (2, 20, 40, 41). The 2019 European Society for the Study of Coeliac Disease (ESsCD) guidelines recommend adding immunosuppressive drugs such as thiopurines (specifically azathioprine or 6-Thioguanine) following a response to steroids, as this may lead to better healing of histological lesions. If the patient responds, annual follow-up with endoscopic exams and biopsies should be performed. If not, dosage of thiopurines should be optimized or the diagnosis of RCD-I should be carefully reconsidered (2).

In RCD-II, steroids are also recommended as first-line therapy (32, 40), being associated with a favourable clinical response (20). Second line therapies generally include multimodality chemotherapy (*e.g.* cladribine, pentostatine, or fludarabine) to eliminate the aberrant RCD-II IELs. If symptoms worsen, high-dose chemotherapy followed by autologous stem cell transplantation (ASCT) is recommended (2, 42–44). The latter shows high response rates (85% of cases) with 4-year overall survival of 66% (45, 46).

The prognosis of RCD varies depending on disease subtype. In general, RCD-II fares much worse than RCD-I (5-year survival rates: 80-95% in RCD-I; 44–58% in RCD-II), due to the severity of the clinical picture and to the higher risk of EATL evolution (21, 25, 47). In fact, RCD-II can be regarded as an aggressive *in situ* T-cell lymphoma of the GI tract (*i.e.* “*in situ* EATL”), closely related and rapidly progressing to overt EATL (48). Malignancies and starvation represent the main causes of death among RCD-II patients (17).

## 3 Pathophysiology of RCD

Over the last decades, several studies have explored the biology and pathophysiology of RCD. It is now clear that RCD-I and RCD-II are very different diseases, sharing a common antigenic trigger and following different pathogenic pathways. This may explain the different epidemiology, clinical features and outcome of RCD-I and RCD-II patients.

### 3.1 Pathophysiology of RCD-I

The pathophysiology of RCD-I is largely unexplored. Like RCD-II, some environmental factors may be associated with RCD-I, such as poor adherence to a GFD (49, 50) and viral infections. The mechanisms of such association are still hypothetical, yet viral infections may increase the production of type I interferon, thus promoting the proliferation of CD8+ T-cells and natural killer (NK) cells, either directly or via the induction of IL-15. This, in turn, may foster anti-gluten immunological reactions, prompting their evolution to a fully autoimmune (*i.e.* gluten-independent) disease (26, 51). This scenario may exist also for other environmental and/or host-related factors, but further studies are needed to investigate this possibility.

### 3.2 Pathophysiology of RCD-II

**Figure 2:**
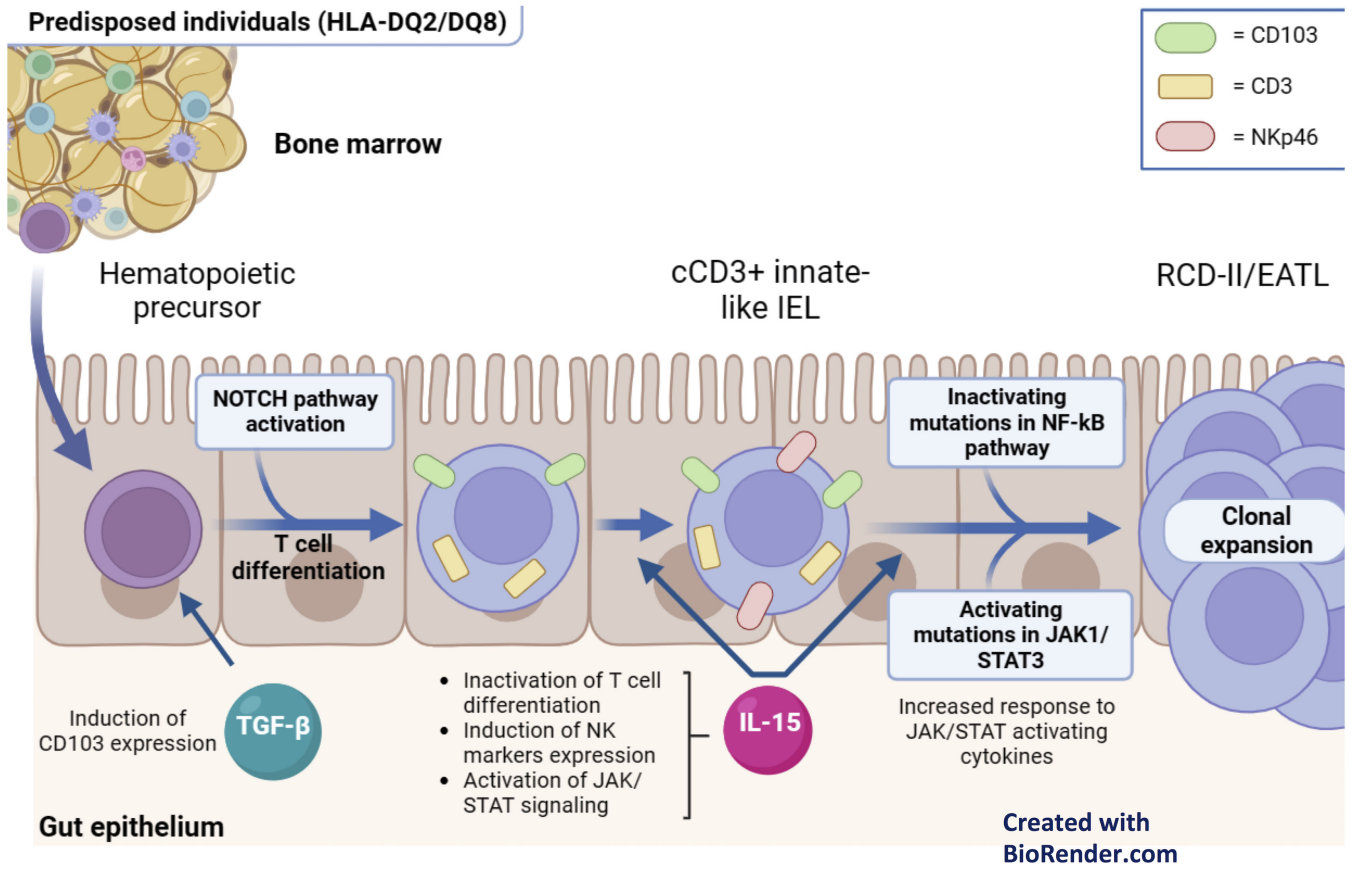
Pathophysiology of RCD-II and EATL. Hematopoietic precursors migrate into the gut epithelium and initiate T cell differentiation in response to NOTCH1 signals. Additionally, they express CD103 in response to TGF-b. In the presence of IL-15, which is overexpressed in the lamina propria and intestinal epithelium of patients with active CD and RCD, these cells inactivate T cell differentiation and express NK cell markers. As a result, these innate-like lymphocytes manifest at the same time T cell (cCD3+) and NK (NKp46+) features. Subsequently, their clonal expansion is driven by gain-of-function somatic mutation in the JAK1/STAT3 pathway, which enhance their responsiveness to IL-15, and/or by loss-of-function mutations in negative regulators of the NF-Kb signaling. .

In the last years, several studies have disclosed genetic and immunological determinants of RCD-II and EATL (**Figure 2**).

As for the genetic factors, both RCD-II and EATL are strongly associated with homozygosity of HLA-DQ2. This is reported in 44-65% of RCD-II and in 53.3% of EATL, while it is documented in only 25.5% of RCD-I and in 20.7% of uncomplicated CD (52). Besides HLA haplotypes, RCD-II and EATL are frequently associated with the *rs7259292* single nucleotide polymorphism (SNP) of the *MYO9B* (53). Progression to RCD-II has also been linked to specific SNPs on chromosome 7 (*rs2041570*) (54). The biological bases of such genetic associations are still under investigation.

As for the immunological determinants, recent studies have shown a link between the neoplastic IELs of RCD-II/EATL and innate-like lymphocytes (ILLs) of normal intestinal mucosa. ILLs are a unique immune cell subset, deriving from immature hematopoietic precursors that migrate into the gut epithelium, start T-cell differentiation in response to NOTCH1 signals, and underwent cell fate reprogramming after IL-15 exposure. Like neoplastic IELs of RCD-II/EATL, ILLs manifest dual T and NK cell traits (55), lack sCD3 and express cCD3, CD103 and various NK receptors (56).

In RCD-II and EATL, the clonal expansion of ILLs is likely driven by somatic gain-of-function mutations of the JAK-STAT pathway (*i.e. JAK1* and *STAT3* mutations), which enhance response to several cytokines, including IL-15. This is overexpressed in the intestinal mucosa of active CD and RCD and stimulates the proliferation of mutated ILLs (50, 51). Besides the JAK-STAT pathway, RCD-II and EATL bear frequent loss-of-function mutations in negative regulators of the NF-kB pathway (*i.e. TNFAIP3* and *TNIP3*) (57). This supports the expansion of ILL clones, since the NF-kB pathway enhances JAK-STAT-regulated transcriptional programs (58). Finally, the NF-kB and JAK-STAT pathways are sustained by the production of TNFα by IELs (59), by the secretion of several cytokines from gliadin-specific CD4+ T cells (60) and by extra-cellular signals mediated by Smad7 (61).

In RCD, the pathogenic role of IL-15 spans well beyond the pro-survival signals provided to neoplastic IELs. IL-15 is indeed largely responsible of GFD-independent mucosal damage and severe villous atrophy, since it induces a NK-like cytotoxic phenotype in IELs (55). In keeping with this, recent studies have documented the expression of NKp46 (a NK-related marker) in most IELs of RCD-II, in 83% of EATL and 100% of monomorphic epitheliotropic intestinal T-cell lymphomas (MEITL), suggesting a shared biology for these conditions (see paragraph 5.4) (62). Thus, besides its pathogenic relevance, NKp46 may serve as a new marker for the differential diagnosis between RCD-I and RCD-II and might represent a target for future therapies in RCD-II/EATL and MEITL (**Figure 2**). Similarly, the identification of the pathogenic role of IL-15 may lead to the future use of anti-IL-15 monoclonal antibodies (2).

## 4 Histopathology of RCD

Histology is the mainstay of RCD diagnosis. Despite this, the microscopic changes of RCD are not entirely specific and overlap with a broad range of conditions, which must be taken into account when facing long-lasting villous atrophy with or without increased IELs. In the following paragraphs the key histological findings of RCD-I and RCD-II will be addressed, specifically considering the differential diagnosis of these entities.

### 4.1 Pathological features and differential diagnosis of RCD-I

**Figure 3:**
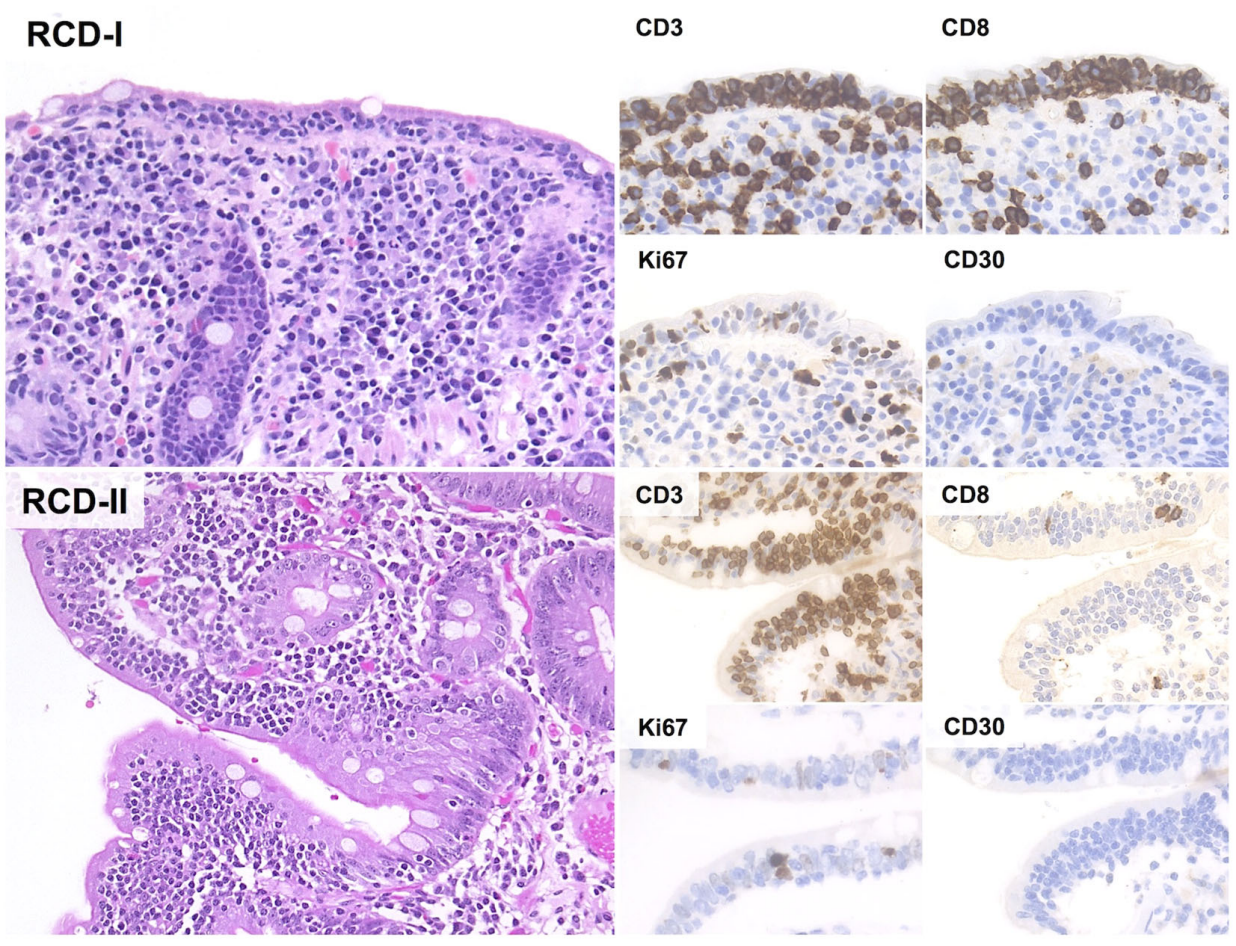
Histological and Immunohistochemical features of RCD-I and RCD-II. In both RCD-I and RCD-II, microscopic examination shows increased IELs without significant cytological atypia. The Ki67 proliferation index is low and CD30 immunostain is negative. However, IELs of RCD-II have an aberrant immunophenotype with negativity for both CD4 and CD8 (H&E and immunoperoxidase stains; original magnification 20x).

The microscopic changes of RCD-I are virtually indistinguishable from those of uncomplicated CD (48). These include villous atrophy, crypt hypertrophy and increased IELs (>25 IELs/100 epithelial cells) with regular expression of pan-T cell markers and positivity for CD8 (48) (**Figure 3**). Molecular studies usually show polyclonal *TCR* gene rearrangements (32, 48, 63).

The differential diagnosis of RCD-I encompasses RCD-II and several CD-unrelated enteropathies, including autoimmune enteropathy (AIE), drug-induced enteropathy (DIE), common variable immunodeficiency (CVID)-associated enteropathy, tropical and collagenous sprue, Giardiasis and Crohn’s disease. While the differential diagnosis with RCD-II relies on IELs phenotype (see below), distinction from other enteropathies requires integration of clinical-epidemiological, histological and laboratory data (**Table 2**) (64).

**Table 2:** Differential diagnosis of RCD-I.

Condition	Key features supporting the differential diagnosis with RCD-I	
	Clinical-laboratory features	Histological features
Autoimmune enteropathy	• Positivity for anti-gut epithelial cell Ab	• Mildly increased IELs • Crypt apoptotic bodies, decreased Paneth and goblet cells
Drug-induced enteropathy	• Improvement after drug withdraw	• Mildly increased IELs • Thick band of sub-epithelial collagen (in Olmesartan-associated enteropathy)
Common variable immunodeficiency-associated enteropathy	• Clinical history of recurrent infections Documentation of severe hypogammaglobulinemia	• Mildly increased IELs and variable VA • Paucity of plasma cells, florid follicular hyperplasia, crypt apoptotic bodies and/or neutrophil infiltration
Tropical sprue	• History of travels to the tropics	• Mildly increased IELs and lower degrees of VA •‘ Decrescendo pattern' of IELs, increased eosinophils in the LP
Giardiasis	• Positivity for Giardia-specific stool Ag • PCR positivity for Giardia *spp* • Identification of cysts/trophozoites in fresh faeces	• Identification of cysts/trophozoites in biopsy samples
Indolent T-cell lymphoma of the GI tract	• Detection of clonal TCR gene rearrangements	• No significantly increased IELs • Striking LP involvement
Collagenous sprue	—*	• No increase of IELs • Thick (≥12 μm) sub-epithelial band of collagen • Severe crypt atrophy with marked reduction of duodenal mucosa thickness
Crohn’s disease	• History of Crohn’s disease • Extensive GI involvement • Luminal narrowing/mucosal cobblestoning on endoscopy	• Mildly increased IELs in a ‘decrescendo pattern’ • Mucosal erosions, crypt distortion, epithelioid granulomas, focal cryptitis/endocryptitis and sub-mucosal extension of the inflammatory process

*No clinical-laboratory features help in the differential diagnosis between RCD-I and Collagenous sprue. Ab, antibodies; Ag, antigens; GI, Gastrointestinal; IELs, Intraepithelial lymphocytes; LP, Lamina propria; PCR, polymerase chain reaction; RCD-I, Refractory celiac disease type I; TCR, T-cell receptor; VA, villous atrophy.

Unlike RCD-I, AIE usually affects young adult patients (mean age at diagnosis: 44 years) with slight male predominance (M:F ratio= 1.5) (65). Histologically, RCD-I and AIE disclose similar degrees of villous atrophy, but IELs are usually lower in the latter. Crypt apoptotic bodies, loss or marked reduction of Paneth and goblet cells support the diagnosis of AIE (65). Finally, AIE is invariably associated with positivity for anti-gut epithelial cell (*i.e.* anti-enterocyte or anti-goblet cell) auto-Ab, which are never documented in RCD (66).

Among DIE, Olmesartan-associated enteropathy (OAE) and Mycophenolate Mofetil-associated enteropathy (MMAE) closely mimic RCD-I (67). OAE is an extremely rare condition (68, 69), characterized by CD-like symptoms after long-lasting consumption of Olmesartan (69). A similar enteropathy has been associated with Valstartan and Irbesartan use (70, 71). The endoscopic and histological findings of OAE may be indistinguishable from RCD-I, although a thick band of collagen may occasionally be observed in the former (69). MMEA presents with persistent diarrhea and villous atrophy due to inhibition of enterocyte proliferation. Like other DIE (*i.e.* Methotrexate and Azathioprine-induced enteropathy), MMAE has low numbers of IELs, supporting the differential diagnosis with RCD-I (72–74). In all such cases, the diagnosis of DIE is definitely confirmed by trials of drug withdraw after careful consideration of ongoing and prior treatments (67).

CVID-associated enteropathy may mimic CD/RCD-I both clinically and histologically (75). Small bowel biopsies reveal a moderate increase in IELs (75.6% of cases) with variable villous atrophy (31.2%-87.5% of cases) (75–77). Distinctive morphological features of CVID-associated enteropathy include extreme paucity of plasma cells, florid follicular hyperplasia in the lamina propria, crypt apoptotic bodies and/or neutrophil infiltration (75). The documentation of severe hypogammaglobulinemia and the history of repeated infections further support the diagnosis (78).

Tropical Sprue is a malabsorption syndrome likely caused by long-lasting infections contracted by natives or travelers to the tropics (79). Compared to CD/RCD-I, tropical sprue features lower degrees of villous atrophy, less numerous IELs, a ‘decrescendo pattern’ of IELs (*i.e.* main location in the villous basal third and in crypt epithelium), and increased eosinophils in the lamina propria. These findings and the history of travels to the tropics support the diagnosis (80).

Giardiasis is another infective enteropathy caused by *Giardia lamblia* that can mimic CD. Giardiasis may display a wide histological spectrum with variable villous atrophy and IELs, therefore its diagnosis relies on the documentation of Giardia-specific stool antigens, on PCR studies for Giardia-specific nucleic acids or on the microscopic detection of cysts/trophozoites in fresh faeces or biopsy samples (64, 81).

Careful histological evaluation also contributes to the differential diagnosis between RCD-I and collagenous sprue. This is indeed characterized by a thick (≥12 μm wide) sub-epithelial band of collagen, entrapping the blood vessels and stromal cells of the lamina propria. In collagenous sprue, villous distortion is usually accompanied by severe crypt atrophy, resulting in a markedly reduced thickness of duodenal mucosa. These findings and the lack of increased IELs favor the diagnosis of collagenous sprue (82).

Indolent T-cell lymphoproliferative disorders of the GI tract must be included in the differential diagnosis of CD/RCD, as they manifest with variable villous atrophy and crypt hypertrophy. Striking involvement of the lamina propria, lack of increased IELs and detection of clonal *TCR* gene rearrangements support the diagnosis of these conditions (48, 64).

Finally, duodenal involvement by Crohn’s disease may closely mimic CD/RCD-I. In such cases, thorough clinical-pathological correlations are mandatory to make the correct diagnosis (83). On clinical grounds, Crohn’s duodenitis is usually associated with more conventional ileal and colonic presentations. On histology, IELs are usually fewer and mainly arranged in a ‘decrescendo pattern’ (84). Mucosal erosions, crypt distortion, epithelioid granulomas, focal cryptitis/endocryptitis and sub-mucosal extension of the inflammatory process further support Crohn’s disease (1, 85).

### 4.2 Pathological features and differential diagnosis of RCD-II

Although RCD-I and RCD-II have overlapping morphology, they are biologically distinct disorders with different malignant potential. RCD-II is indeed a pre-lymphomatous condition characterized by clones of phenotypically aberrant IELs. Phenotypic aberrancies in RCD-II are defined by negativity for sCD3 and CD8, with positivity for cCD3 (48) (**Table 1**; **Figure 3**). Of note, clonal *TCR* rearrangements are documented in most RCD-II, but they are neither specific nor required for the diagnosis. In fact, clonal TCR rearrangement can be detected in a minority of CD and RCD-I patients (63). Clonal testing can also provide false negative results when atypical clones are small (63, 86, 87) and/or have incomplete/non-functional *TCR* rearrangements (70% of RCD-II) (63, 88, 89).

Phenotypic aberrancies in RCD-II can be documented by either flow cytometry or immunohistochemistry (IHC). As a general rule, flow cytometry is more sensitive and accurate, although it is not as widely applicable as IHC (48). As such, both techniques can be used to make a diagnosis of RCD-II, but different thresholds for aberrant IELs should be considered (*i.e.* ≥20% of total IELs for FC; ≥50% of total IELs for IHC) (87, 90).

Morphological assessment of duodenal biopsy in RCD-II shows marked villous atrophy, usually at a greater degree than RCD-I (moderate/severe villous atrophy: 96% of RCD-II and 50% of RCD-I) (20), together with a predominantly intra-epithelial infiltrate of atypical lymphocytes. Minimal sub-epithelial infiltration is frequently observed, constituting up to 20% of lymphocytes in the lamina propria (91). Notably, atypical IELs can be detected all throughout the GI tract, as well as in peripheral blood, mesenteric lymph nodes, lung parenchyma, skin and bone marrow (21, 91–94). In keeping with this, lymphocytic gastritis and lymphocytic colitis with abnormal IELs are reported in roughly 30-50% of RCD-II (21). Such widespread distribution also provides an explanation to the extra-intestinal presentations of EATL, which constitute up to one-third of cases (95).

Phenotypically, IELs of RCD-II lack CD4, CD8, sCD3 and TCRαβ/TCRγδ, while retaining CD7, CD103 and cCD3 expression (93). CD30 is characteristically negative and its expression suggests evolution to EATL (96). Alternative phenotypes are occasionally seen, including positivity for sCD3, CD8, TCRαβ and/or TCRγδ (48).

The differential diagnosis of RCD-II mainly includes RCD-I and EATL. Distinction from RCD-I relies primarily on IEL phenotyping, while the differential diagnosis with EATL is more challenging. In fact, RCD-II and EATL represents two ends of a biological continuum and a diagnosis of overt EATL should only be considered when the neoplastic population massively invades the duodenal/small intestinal wall, with clear-cut evidence of tumor lesions, bowel perforation or strictures (see paragraph 5.4).

## 5 EATL: biology and clinical-pathological features

### 5.1 Definition and pathobiology of EATL

EATL is an extremely aggressive peripheral T-cell lymphoma (PTCL), arising from IELs of the small bowel and representing the invasive form of RCD-II. In line with this, EATL and RCD-II share several pathophysiological features, including a common genetic background (*i.e.* homozygosity for HLA-DQ2; common allelic variants of *MYO9B* gene) (52, 53) and overlapping mutations in the JAK-STAT and NF-kB pathway (57). Additional events in the pathogenesis of EATL include oncogenic mutations in *TET2*, *POT1*, *DDX3X*, *PRDM1/BLIMP1* and *KMT2D* (57, 97), deletions of 16q12.1 and gains of 1q, 5q and 9q (98). All of this contributes to the acquisition of an aggressive phenotype, whereby intra-mucosal lymphocytes of RCD-II undergo uncontrolled proliferation, invading the intestinal wall, disseminating throughout the GI tract and, ultimately, to extra-intestinal sites.

### 5.2 Epidemiology of EATL

Despite being the most common intestinal T-cell lymphoma in Western countries, EATL is an exceedingly rare disease with a reported incidence of 0.2-1.0/1.000.000/year. It accounts for 5% of all GI lymphomas (99–101) and for only 3% of PTCLs (102). Virtually all cases arise in the setting of CD and the geographic distribution of the disease likely reflects the higher prevalence of CD in the Western world (48). Depending on the time relationship with CD, two forms of EATL are reported, namely primary EATL (*i.e.* EATL diagnosed concurrently with CD) and secondary EATL (*i.e.* EATL arising in patients with prior diagnosis of CD or RCD-II).

EATL affects adult to elderly patients (median age at diagnosis: 61 years) (95) and likely develops several months to years after the onset of pre-malignant IEL clones, which may remain clinically silent for a long time. In keeping with this observation, up to 50% of EATLs arise in the setting of RCD-II (95), thus confirming a tight connection between the two entities.

### 5.3 Clinical-prognostic features of EATL

**Figure 4:**
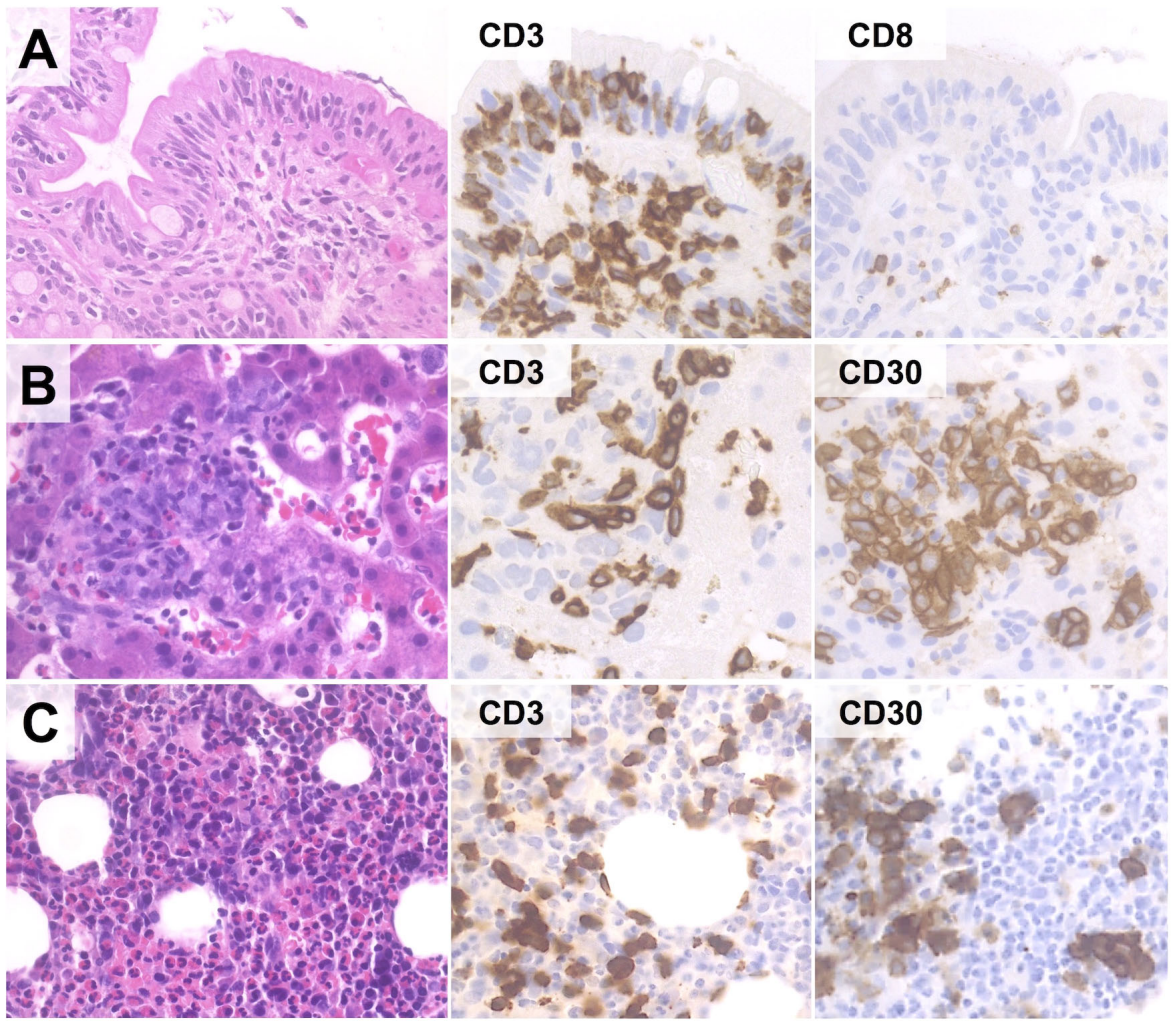
Extra-instestinal presentation of EATL. In this case (67-year old female with history of RCD-II), duodenal biopsy discloses only IELs with aberrant phenotype (CD3+/CD4-/CD8-/CD30-) **(A)**. However, liver **(B)** and bone marrow **(C)** biopsies reveal an atypical lymphoid infiltrate comprising numerous CD30+ blasts, suggesting the diagnosis of extra-intestinal progression to EATL. (H&E and immunoperoxidase stains; original magnification 40x).

Clinically, EATL presents with small intestine lesions in about 90% of cases, the jejunum being most frequently involved. Multifocality is observed in 30-55% of cases and advanced-stage disease (Lugano stage II_2_-IV) is present in about half of the patients (95, 102). As previously reported, a subset of cases presents primarily in extra-intestinal sites, such as the spleen, the lung and the liver (95) (**Figure 4**). Typical signs and symptoms include abdominal pain, weight loss and diarrhea (102, 103) perforations, obstructions and/or GI bleeding (95). B symptoms (besides weight loss) are reported in one third of the patients (48). Laboratory tests are non-specific with anemia, high LDH and B2M levels and low serum albumin due to starvation (30, 95). Imaging studies often reveal enteric strictures, perforations or mass lesions, as well as mesenteric adenopathies and/or splenomegaly (27, 39). Although these findings are highly suggestive of EATL in the setting of CD, a definite diagnosis is only posed by histological evaluation of endoscopic biopsies or resection specimens.

At present, high-dose chemotherapy followed by ASCT is the mainstay of treatment (104). Unfortunately, only a minority of patients can be treated with such an aggressive approach and the outcome remains poor (5-year OS: 11-20%) (105, 106). To refine the prognostic stratification of patients, a multi-parametric score has been recently proposed by integrating the International Prognostic Index and the presence of B-symptoms (*i.e.* EATL Prognostic Index [EPI]). The EPI identifies the following risk groups: (i) low-risk EATL (IPI score <2 and no B-symptoms; median OS: 34 months); (ii) intermediate-risk EATL (IPI score ≥2 and no B-symptoms; median OS: 7 months); and (iii) high-risk EATL (presence of B-symptoms irrespective of IPI score; median OS: 2 months) (107). In addition to EPI, the time relationship between CD and EATL likely influences outcome, in that primary EATL seems to fare better than secondary (*i.e.* post-RCD-II) disease (5-year OS: 60% versus <5%) (95). These prognostic parameters and the recent identification of new therapeutic targets (*e.g.* CD30 and NKp46 expression on neoplastic cells; IL15 in the tumor microenvironment) will hopefully contribute to improve patient management (108, 109).

### 5.4 Pathology and differential diagnosis of EATL

**Figure 5:**
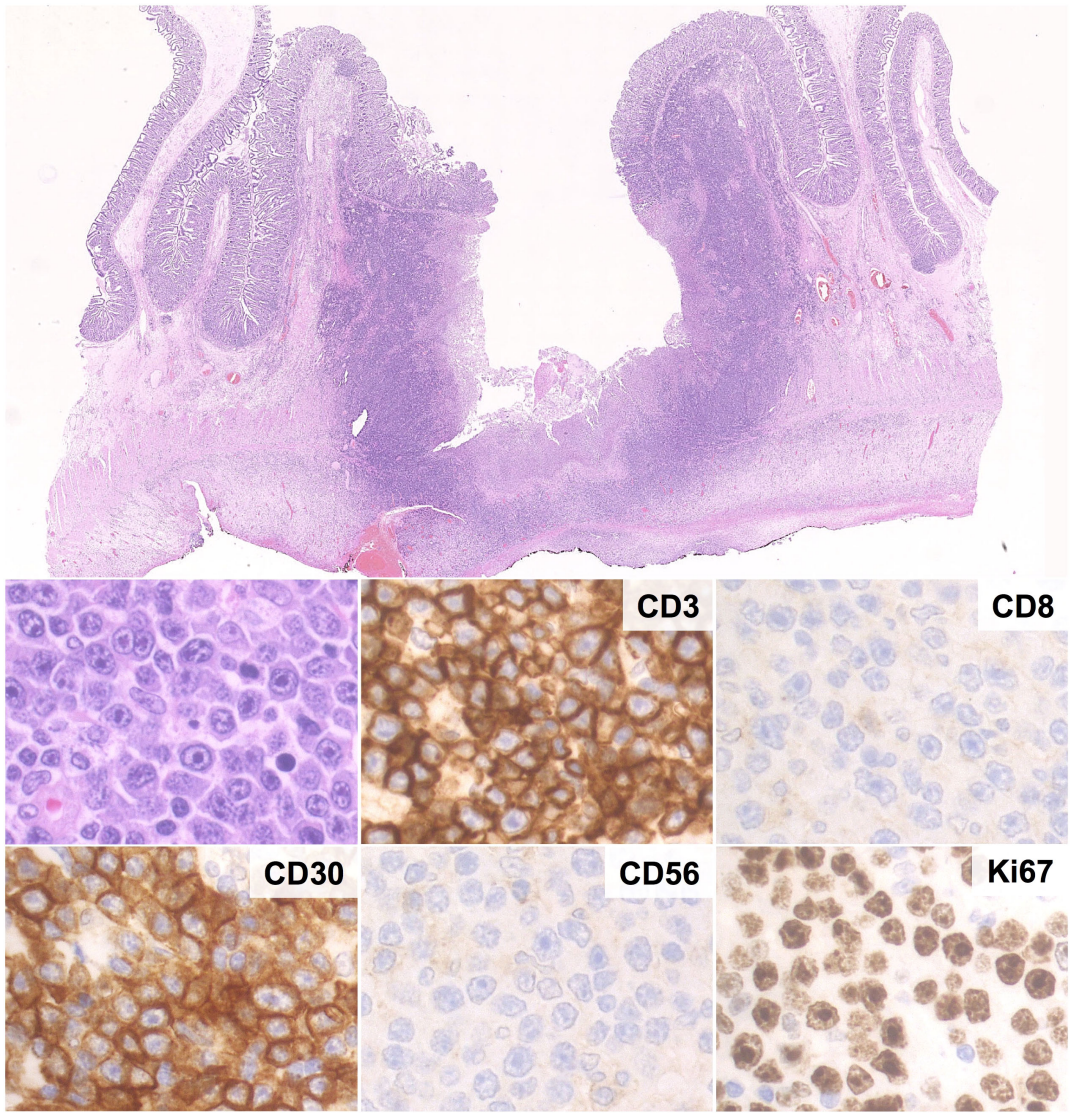
Histological and Immunohistochemical features of EATL. Microscopic examination shows a massive infiltration of the intestinal wall by sheets of CD3+ cells with immunoblastic morphology, high prolifration index, diffuse positivity for CD30 and negativity for CD8 and CD56. (H&E and immunoperoxidase stains; original magnification 1, 25x and 40x).

Histologically, EATL is characterized by a diffuse infiltrate of atypical T-cells in a rich inflammatory background of histiocytes, plasma cells and granulocytes. Reactive cells may be as many as to obscure the neoplastic population, which consists of medium to large cells with pleomorphic, immunoblastic or anaplastic morphology (95, 102, 110). The infiltrate may be confined to the mucosa/submucosa or may extend throughout the intestinal wall (**Figure 5**); angioinvasion and angiodestruction can also be observed (48). The adjacent mucosa usually discloses features of CD/RCD-II (102, 110).

The phenotype of EATL largely recapitulates that of RCD-II cells, with combined expression of T-cell and NK-cell markers (*i.e.* positivity for cCD3, CD7, NKp46 and CD103; variable expression of CD2; negativity for CD4, CD5, CD8, CD56, ALK, EBER and TCRαβ/TCRγδ) (62, 102, 111). Neoplastic cells usually show positivity for cytotoxic markers (TIA1, granzyme B, perforin), a high proliferation index (>50%) and CD30, mostly in cases with anaplastic/immunoblastic morphology (95, 102). A minority of cases shows aberrant phenotypes with positivity for CD8 (25% of cases) and/or TCR proteins (48). Molecular analyses disclose clonal *TCR* rearrangements in most cases (57).

The differential diagnosis of EATL mainly includes RCD-II and MEITL. As previously outlined, distinction from RCD-II relies on the degree of infiltration by neoplastic cells, which is usually massive in EATL and limited to the lamina propria in RCD-II. However, separating early-stage (*i.e.* mucosa/submucosa-limited) EATL from RCD-II may be matter of subjectivity, especially on small biopsy samples. In such cases, the diagnosis of EATL should be favored in presence of B symptoms, abdominal masses, or specific phenotypic findings (*e.g.* positivity for CD30; high Ki67 index).

Likewise, distinction from MEITL relies on clinical, morphological, phenotypic and genetic criteria. Unlike EATL, MEITL is rarely associated with CD/RCD-II (98, 112, 113) and consists of a monomorphic population of small-to-medium lymphocytes with little inflammatory background and sharp epitheliotropism (48). The phenotype of MEITL also differs from EATL, in that the neoplastic cells are positive for CD8, CD56, SYC and TCR proteins (usually of γδ type). Despite these differences, EATL and MEITL likely share a common origin from intestinal IELs, as indicated by their clear-cut epitheliotropism, by CD103 and NKp46 expression (62) and by similar activating mutations in the JAK-STAT pathway (114, 115). Unlike EATL, however, MEITL shows frequent *SETD2* alterations, which may support the correct diagnosis (116).

Distinction of EATL from other primary GI lymphomas (*i.e.* aggressive B-cel lymphomas; intestinal T-cell lymphoma NOS; indolent T-cell lymphoproliferative disorders of the GI) and from GI involvement by systemic PTCL is usually straightforward and relies on a combination of morphology, phenotypic studies and clinical correlations.

## 6 Conclusions

Over the last decades, a batter characterization of the biology of RCD and EATL has improved our knowledge of these conditions. RCD-I and RCD-II are distinct disorders stemming from a disease initially driven by abnormal T-cell immune responses against gluten-derived peptides in genetically susceptible individuals. In particular, RCD-I represents a gluten-independent dysimmune reaction of the small bowel, while RCD-II can be regarded as an aggressive *in situ* T-cell lymphoma with high risk of EATL progression. In keeping with this view, several studies have highlighted the complex pathogenesis and kinship of RCD-II and EATL. All of this has been formally acknowledged also by the 2022 WHO and ICC classifications of lymphoid tumors, which include both EATL and RCD-I/RCD-II in the list of intestinal T-cell lymphoproliferative disorders (48).

Despite these achievements, the diagnosis of RCD and EATL remains challenging and the prognosis of RCD-II and EATL is poor. New molecular targets for tailored therapies will hopefully compensate for such dismal outcome. For the time being, the proper recognition and management of RCD and EATL relies on a high degree of suspicion, on careful differential diagnoses, and on the collaboration of gastroenterologists, hematologists and pathologists with specific expertise on GI lymphomas and dysimmune disorders. This teamwork still represents the best strategy for any further development on these conditions and for the appropriate management of patients.

## Author contributions

FS: Writing – original draft. MP: Writing – original draft, Supervision, Writing – review & editing. FPe: Writing – review & editing. VA: Writing – review & editing. AD: Supervision, Writing – review & editing. FPi: Writing – review & editing. ES: Writing – review & editing. FZ: Supervision, Writing – original draft. MF: Supervision, Writing – original draft.
